# Tomato Powder Modulates NF-*κ*B, mTOR, and Nrf2 Pathways during Aging in Healthy Rats

**DOI:** 10.1155/2019/1643243

**Published:** 2019-01-02

**Authors:** Kazim Sahin, Cemal Orhan, Mehmet Tuzcu, Hakki Tastan, Birdal Bilir, Nurhan Sahin, Deniz Aslar Oner, Omer Kucuk

**Affiliations:** ^1^Department of Animal Nutrition, Faculty of Veterinary Medicine, Firat University, Elazig, Turkey; ^2^Department of Biology, Faculty of Science, Firat University, Elazig, Turkey; ^3^Department of Biology, Faculty of Science, Gazi University, Ankara, Turkey; ^4^Winship Cancer Institute of Emory University, Atlanta, GA, USA

## Abstract

**Purpose:**

In the present study, we aimed to investigate the effects of tomato powder (TP) on glucose and lipid metabolism, as well as oxidative stress and the NF-*κ*B, mTOR, and Nrf2 pathways during the aging process in healthy rats.

**Methods and Results:**

Male Wistar rats were randomly assigned to four groups as follows: (i) Control group 1 (*n*=15, 3-week old): rats were fed standard diet for 7 weeks; (ii) TP group 1 (*n*=15, 3-week old): rats were fed standard diet supplemented with TP for 7 weeks; (iii) Control group 2 (*n*=15, 8-week old): rats were fed standard diet for 69 weeks; and (iv) TP group 2 (8-week old): rats were fed standard diet supplemented with TP for 69 weeks. TP supplementation significantly reduced the hyperglycemia, hypertriglyceridemia, and hypercholesterolemia and improved liver function and kidney function in 77-week old rats compared with the control animals (*P* < 0.05). In addition, TP significantly decreased the serum and liver MDA levels (*P* < 0.003 and *P* < 0.001, respectively) while increasing the activities of liver SOD (*P* < 0.001), CAT (*P* < 0.008), and GPx (*P* < 0.01) compared with the control groups in both 10-week-old and 77-week-old rats (*P* < 0.05). Age-related increases in phosphorylation of NF-*κ*Bp65, mTOR, 4E-BP1, and P70S6K were observed in livers of 77-week-old rats compared to those of 10-week-old rats (*P* < 0.001). TP supplementation decreased the expression of NF-*κ*Bp65 and activation of mTOR, 4E-BP1, and P70S6K in livers of 77-week-old rats compared to the control animals. Moreover, TP supplementation significantly elevated Nrf2 expression in livers of both 10-week-old and 77-week-old rats (*P* < 0.05).

**Conclusion:**

TP ameliorates age-associated inflammation and oxidative stress through the inhibition of NF-*κ*Bp65, mTOR pathways, and Nrf2 activation may explain the observed improvement in glucose and lipid metabolism as well as the improved liver and kidney functions.

## 1. Introduction

Aging is a complex and multifactorial biological process characterized by a progressive loss of structural integrity and physiological function. Although the cellular and molecular mechanisms of aging still remain poorly understood, an increasing body of evidence shows that oxidative stress and inflammation, described by elevated levels of lipid peroxidation and proinflammatory cytokines, are involved in the aging process and the development of age-related diseases [1–3]. Oxidative stress can lead to changes in cell proliferation, apoptosis, and angiogenesis as well as genetic instability, including increased DNA damage, alterations in DNA repair, and aberrant DNA methylation [4, 5]. Increased levels of proinflammatory and oxidative stress markers, including nuclear factor-kappaB (NF-*κ*B), interleukin 6 (IL-6), tumor necrosis factor-*α* (TNF-*α*), and malondialdehyde (MDA), have been associated with age-related diseases, such as cardiovascular disease, type 2 diabetes, osteoporosis, and autoimmune diseases [5].

The transcription factor NF-*κ*B is one of the key modulators of inflammation that is regulated by several signal transduction pathways, including mammalian target of rapamycin (mTOR), thereby controlling cell growth, metabolism, proliferation, survival, aging, synaptic plasticity, and memory [6]. NF-*κ*B, which plays important roles in the immune system, is ubiquitously expressed and retained inactive by inhibitors of NF-*κ*B (I*κ*B) in the cytoplasm of unstimulated cells [7]. Upon activation by growth factors and cytokines, I*κ*B is phosphorylated and ubiquitinated, and NF-*κ*B translocates to the nucleus, where it alters expression of specific target genes involved in a wide variety of cellular functions, including apoptosis, neoplastic transformation, proliferation, invasion, metastasis, and inflammation [7]. It has been reported that production of reactive oxygen species (ROS), which in turn lead to oxidative stress, can activate NF-*κ*B signaling, resulting in perturbation of cellular homeostasis [8]. NF-*κ*B has been implicated in inflammatory responses related to aging and age-related diseases, including cancer, obesity, and diabetes [8, 9].

The nuclear factor erythroid 2-related factor 2 (Nrf2) transcription factor, which is a downstream target of NF-*κ*B, has been shown to mediate both inducible and constitutive expression of antioxidant response element- (ARE-) regulated genes, including those coding for a number of antioxidant proteins and phase II detoxifying enzymes that defend the cell against electrophilic stressors and oxidative insults [9–11]. Previous studies have reported a reduction of approximately 50% in nuclear Nrf2 levels and ARE binding in livers of aged rats [10, 12].

The mammalian target of rapamycin (mTOR) pathway has been shown to play important role in human diseases, and inhibition of this pathway extends life span in model organisms [13]. mTOR forms two functionally distinct complexes, mTOR complex 1 (mTORC1) and mTOR complex 2 (mTORC2) [14]. mTORC1 regulates many cellular processes including cell growth, ribosomal biogenesis, protein translation, and autophagy through phosphorylation of substrates, including S6 kinase 1 (S6K1) and eukaryotic translation initiation factor 4E binding protein 1 (4E-BP1) [15, 16].

Several studies have reported that antioxidants, including phytochemicals, extend the average life span and have a tendency to reduce body weight, lipofuscin, tumor formation, and autoimmune diseases [1]. Tomato phytochemicals include a large amount of lycopene (80–90%) as well as *β*-carotene (7–10%) and small amounts of *α*-carotene, *γ*-carotene, *ζ*-carotene, lutein, zeaxanthin, neoxanthin, *α*-cryptoxanthin, *β*-cryptoxanthin, phytoene, phytofluene, cyclolycopene, and other beneficial molecules, including vitamin C, vitamin E, and flavonoids [17, 18]. Lycopene exhibits strong antioxidant and anti-inflammatory activities, protecting the cells against inflammation and oxidative damage caused by ROS [19, 20]. Many studies have shown that phytochemicals, including lycopene, offer numerous health benefits such as anti-inflammatory, antioxidant, and reduction in body weight, blood pressure, serum glucose and lipids, immunity enhancement, and increased life span [1, 3, 21, 22]. In association with these properties, lycopene plays an important role in the prevention of certain types of cancer, cardiovascular and degenerative diseases by improving gene regulation, gap-junctional communication, immune function, and hormonal and metabolic pathways [19, 23]. However, the antioxidant and anti-inflammatory roles of tomato in healthy rats are not well characterized. Therefore, in the present study, we aimed at investigating the potential effects of tomato powder (TP) on the oxidative stress as well as regulation of NF-*κ*B, mTOR, and Nrf2 signaling pathways throughout the life span of healthy rats.

## 2. Materials and Methods

### 2.1. Animals

A total of 60 male Wistar rats (30 rats at age of 3 weeks and 30 rats at age of 8 weeks) were obtained from Firat University Research Center (Elazig, Turkey). Animals had free access to water and were fed ad libitum standard diet with or without TP and were maintained on a 12-hour light and 12-hour dark schedule. Room temperature was maintained at 21°C ± 1^o^C and humidity at 50% ± 5%. All procedures for the Care and Use of Laboratory Animals in this study were approved by the Ethics Review Committee of Firat University for Animal Experimentation and were strictly followed.

### 2.2. Experimental Design

Rats were randomly assigned to four groups as follows: (i) Control group 1 (*n*=15, 3-week old): rats were fed standard diet for 7 weeks; (ii) TP group 1 (*n*=15, 3-week old): rats were fed standard diet supplemented with TP for 7 weeks; (iii) Control group 2 (*n*=15, 8-week old): rats were fed standard diet for 69 weeks; and (iv) TP group 2 (8-week old): rats were fed standard diet supplemented with TP for 69 weeks. The trial lasted until 10 weeks of age in groups (i) and (ii) (young rats) while it lasted until 77 weeks of age in groups (iii) and (iv) (older rats). The amount of TP added to the standard diet was determined based on a previous study reporting that addition of TP at 5% per kilogram reduced the development of colorectal cancer in rats [24]. TP consists of 11% crude protein, 4.5% fat, 0.8 mg of lycopene, 0.13 mg of *β*-carotene, 1.73 mg of vitamin C, and 0.07 mg of tocopherol per gram of powder (Oz-Gida A.S., Elazig, Turkey).

At the end of the trial, after an overnight fast, blood samples were collected by cardiac puncture, and all animals were sacrificed by cervical dislocation, followed by removal of tissue samples.

### 2.3. Laboratory Analyses

Serum was separated by centrifuging the blood sample at 3,000 × g for 10 minutes and analyzed for biochemical parameters using an automated analyzer (Samsung LABGEOPT10, Samsung Electronics Co, Suwon, Korea). Reproducibility and accuracy of LABGEOPT10 were documented according to the IVR-PT06 guideline.

Serum and liver MDA levels were measured using HPLC with an LC-20AD pump, SIL-20A autosampler, SPD-20A ultraviolet-visible spectroscopy detector (at C18-ODS-3V and 5 *μ*m with a 4.6 mm × 250 mm column), and a CTO-10ASVP column oven (Shimadzu) as described previously by Sahin et al. [25]. An aliquot of 20 *μ*l of the supernatant isolated from serum or liver homogenate was injected into an HPLC column. Samples were eluted with a mobile phase containing 30 mM of KH_2_PO_4_-methanol (82.5 : 17.5, v/v, pH 3.6) at a flow rate of 1.2 ml/minute. Chromatograms were acquired at 250 nm.

To assess the activities of superoxide dismutase (SOD), catalase (CAT), and glutathione peroxidase (GPx), and liver tissues were homogenized in a mixture of 20 mM of HEPES buffer (N-2-hydroxyethylpiperazine-N'-2-ethanesulfonic acid), 1 mM of ethylene glycol tetraacetic acid, 210 mM of mannitol, and 70 mM of sucrose (pH 7.2) and analyzed by ELISA (Biotek Instruments, Inc., Vermont, USA) using a commercial kit according to the manufacturer's instructions (Cayman Chemical, Ann Arbor, MI, USA).

### 2.4. Western Blot Analysis

The nuclear extract from liver was prepared as described previously by Farombi et al. [26]. For this purpose, liver samples were homogenized in 1 ml of hypotonic buffer A (10 mM HEPES (pH 7.8), 10 mM KCl, 2 mM MgCl2, 1 mM DTT, 0.1 mM EDTA, and 0.1 mM phenylmethylsulfonyl-fluoride (PMSF)). To the homogenates was added 80 *μ*l of 10% Nonidet P-40 (NP40) solution, and the mixture was then centrifuged for 2 min at 14000 g. The supernatant was collected as a cytosolic fraction. The precipitated nuclei were washed once with 500 *μ*l of buffer A plus 40 *μ*l of 10% NP-40, centrifuged, resuspended in 200 *μ*l of buffer C (50 mM HEPES (pH 7.8), 50 mM KCl, 300 mM NaCl, 0.1 mM EDTA, 1 mM DTT, 0.1 mM PMSF, and 20% glycerol), and centrifuged for 5 min at 14800 g. The supernatant containing nuclear proteins was collected and stored at −70 C after determination of protein concentrations.

Western blotting was performed as described previously [25]. Fifty *μ*g of protein was separated on 10% SDS-polyacrylamide gel and transferred onto 0.2 *μ*m nitrocellulose membrane (Sigma, St. Louis, MO). Membranes were incubated with primary antibodies (NF-*κ*Bp65, Nrf2, p-mTOR, p-P70S6K, and p-4E-BP1) (1 : 1,000; Abcam, Cambridge, UK). The mouse monoclonal antibody against *β*-actin (Abcam, Cambridge, UK) was used as normalization control. Signals were quantitated using the ImageJ analysis system (National Institute of Health, Bethesda, USA).

### 2.5. Statistical Analysis

The continuous variables (molecular biology data) were analyzed by ANOVA using the PROC GLM procedure. Differences among the groups were attained by Tukey multiple comparisons. If the *p* value is less than 0.05, the difference is considered to be statistically significant.

## 3. Results

In the present study, we investigated the effects of TP supplementation on serum glucose, lipid profile, liver enzymes, and renal function in rats at two different ages. As shown in Table 1, the levels of serum glucose, total cholesterol (T-C), triglyceride (TG), aspartate aminotransferase (AST), alanine aminotransferase (ALT), creatinine (Cr), and blood urea nitrogen (BUN) significantly increased in older rats (10-week-old animals versus 77-week-old animals) (*P* < 0.05). In 77-week-old rats, TP intervention reduced hyperglycemia, hypercholesterolemia, and hypertriglyceridemia. However, TP supplementation of 10-week-old rats had no effect on these biochemical markers in comparison with the control animals at this age (*P* > 0.05). We did not measure the initial and final body weights.

As shown in Table 2, compared to 10-week-old rats, in 77-week-old rats, serum and liver MDA levels increased (1.31 vs. 2.76 and 4.60 vs. 7.72, respectively; *P* < 0.05), whereas the activities of liver enzymes, SOD (184.07 vs. 123.21), CAT (265.88 vs. 204.38), and GPx (33.26 vs. 15.55) decreased (*P* < 0.05). It is of note that TP supplementation reduced the serum and liver MDA levels (*P* < 0.003 and *P* < 0.001, respectively) while it increased the activities of SOD (*P* < 0.001), CAT (*P* < 0.008), and GPx (*P* < 0.01) in 77-week-old rats (*P* < 0.05). Moreover, TP exerted similar effects on MDA levels and liver enzymes in 10-week-old animals.

**Figure 1:**
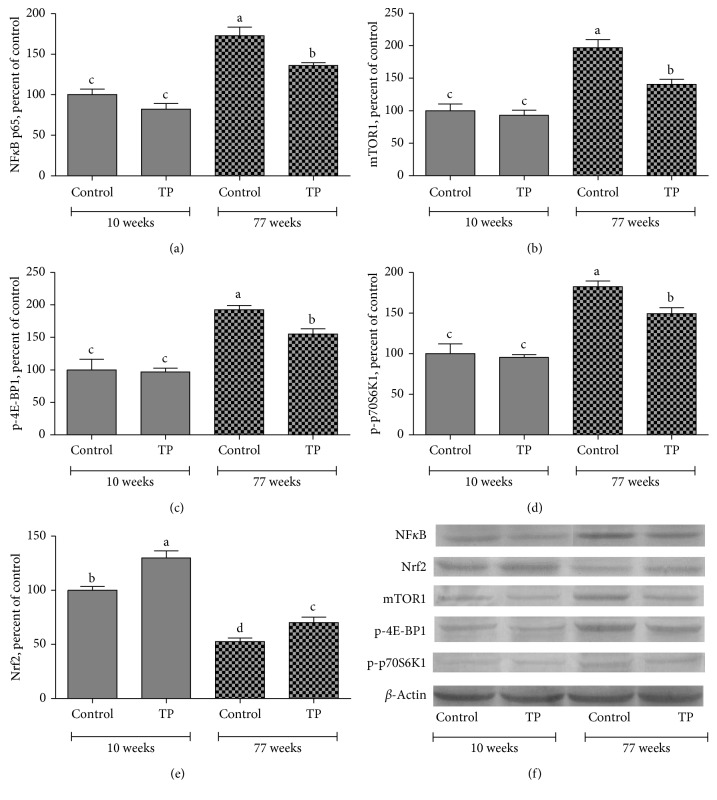
Effects of tomato powder (TP) on expression levels of NF-*κ*B (a), p-mTOR (b), p-4E-BP1 (c), p-p70S6K1 (d), and Nrf2 (e) in livers of healthy rats at ages of 10 weeks and 77 weeks. Intensities of the signals on Western blots were quantiﬁed by densitometric analysis. Data were expressed as a ratio of treatment value to control value, which was set to 100%. Bars represent the standard error of the mean. Western blots were repeated at least three times, and (f) a representative blot was shown. *β*-Actin was included as a protein loading control. Mean values within a bar with different superscript letters were significantly different at *P* < 0.05.

**Figure 2:**
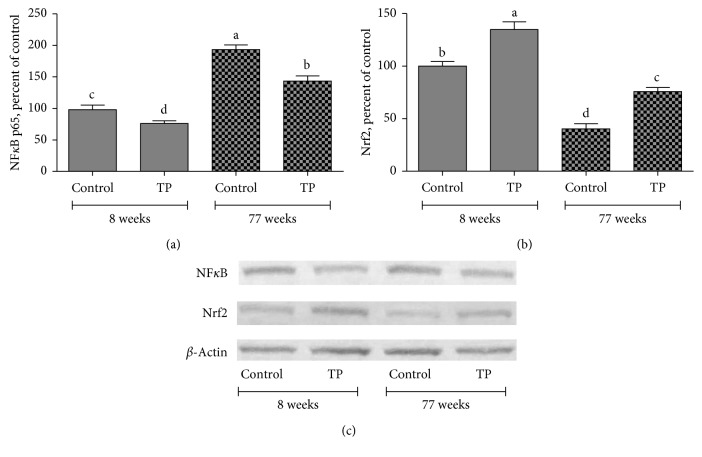
Effects of tomato powder (TP) on expression nuclear levels of NF-*κ*B (A) and Nrf2 (E) in the livers of healthy rats at ages of 10 weeks and 77 weeks. Intensities of the signals on Western blots were quantiﬁed by densitometric analysis. Data were expressed as a ratio of treatment value to control value, which was set to 100%. Bars represent the standard error of the mean. Western blots were repeated at least three times, and a representative blot was shown. *β*-Actin was included as a protein loading control. Mean values within a bar with different superscript letters were significantly different at *P* < 0.05.

Western blot analysis showed that expression levels of NF-*κ*Bp65, p-mTOR, p-4E-BP1, and p-P70S6K1 increased by 72.9%, 97.3%, 82.0%, and 92.6%, respectively, in livers of 77-week-old control rats compared to those of 8-week-old animals (*P* < 0.05; Figures 1(a)–1(d)). We observed that TP supplementation partially reversed the effects of aging on NF-*κ*B, p-mTOR, p-4E-BP1, and p-P70S6K1 proteins through decreasing their expression levels by 21.3%, 28.6%, 18.0%, and 19.2%, respectively (*P* < 0.05; Figures 1(a)–1(d)), in livers of 77-week-old rats. However, TP intervention had no effect on expression levels of these proteins in 10-week-old rats (*P* > 0.05). Additionally, increased age resulted in a 47.4% reduction in the expression level of Nrf2 in the liver (*P* < 0.05; Figure 1(e)). Interestingly, TP supplementation significantly elevated the Nrf2 expression in livers of 77-week-old rats (by 33.2%) as well as those of 10-week-old rats (by 29.8%) (Figure 1(e)). In addition, levels of NF-*κ*Bp65 and Nrf2 were also measured by Western analyses of nuclear extracts from liver homogenates of rats treated with TP. As shown in Figure 2, TP treatment caused decreased NF-*κ*B and increased Nrf2 accumulation in the nuclear fraction.

## 4. Discussion

Tomato and tomato-based food products are good sources of carotenoids, including lycopene, neurosporene, *γ*-carotene, phytoene, and phytofluene. It is noteworthy that lycopene, a natural antioxidant, has been shown to play an important role in cancer prevention through pleiotropic mechanisms [27, 28]. Since humans cannot synthesize lycopene *de novo*, they rely upon the diet as the source of this compound. It has been reported that more than 85% of the dietary intake of lycopene comes from tomato and processed tomato products; lycopene is also obtained from watermelon, pink grapefruit, guava, and papaya [29]. Due to its strong antioxidant activity, lycopene has been extensively investigated [30, 31]. Several studies have reported that lycopene exerts anti-inflammatory effects in the liver [32]. Although the protective effects of lycopene against various types of cancer, obesity, and associated disorders have been well described [33], no studies have yet been reported on the effects of TP on the aging process. Therefore, in the present study, we examined the potential effects of TP on the age-related changes by biochemical analyses as well as its effects on oxidative stress and regulation of the NF-*κ*Bp65, mTOR, and Nrf2 signaling cascades during life span of healthy rats. Our findings showed that serum glucose and lipid levels increased with age. Consistent with previously published studies indicating the antiaging effects of phytochemicals, including resveratrol and EGCG, we observed that TP supplementation improved carbohydrate metabolism, lipid profiles (T-C, TG), kidney and liver functions, lipid peroxidation, and antioxidant enzyme activities in 77-week-old rats [22, 34, 35]. Niu et al. reported that EGCG, an antioxidant supplement, tended to decrease systolic blood pressure and the levels of T-C, TG, LDL-C, and glucose whereas it increased the levels of HDL-C in rats, especially in the late phase of the experiment [22]. Moreover, they showed that EGCG significantly reduced the levels of serum TNF-*α*, IL-6, ROS, and MDA while increasing the levels of SOD and GSH-Px. Of note, Alshatwi et al. demonstrated that TP was more protective than lycopene against lipid peroxidation in rats [31]. Several studies indicated an inverse association between serum lycopene and MDA levels [32, 36]. We have also observed significant reduction in MDA levels with lycopene supplementation (Table 2).

The NF-*κ*B signaling pathway has been associated with oxidative stress and inflammation [37, 38]. It has been reported that oxidative stress and several cytokines are involved in triggering free radical chain reactions, disruption of the functions of organs, including liver, activation of the NF-*κ*B pathway, and elevation of levels of inflammatory markers associated with the aging process and age-related diseases [22, 34]. Of note, several studies have shown that NF-*κ*B signaling is activated during aging [22, 39]. The Nrf2 transcription factor, which is a downstream target of NF-*κ*B, is one of the key antioxidant defense mechanisms that protect cells against oxidative stress [40]. Nrf2 mediates strong antioxidant and cytoprotective responses through binding to antioxidant response elements (AREs), inducing the transcription of genes, including heme oxygenase-1, glutathione peroxidase, glutathione-*S*-transferase, NAD(P)H:quinone oxidoreductase 1, and glutamate-cysteine ligase catalytic subunit [41]. Numerous studies have demonstrated that activation of Nrf2 signaling has the potential to combat oxidative injuries in age-related diseases, particularly those at chronic inflammatory states, improving healthspan [7, 42]. Phytochemicals have been reported to inhibit the nuclear translocation, DNA binding, and transactivation of NF-*κ*B, contributing to the prevention of inflammatory responses in age-related diseases [43]. In the present study, we demonstrated for the first time that TP supplementation significantly reduced the expression level of NF-*κ*Bp65 while inducing the Nrf2 expression in the liver of healthy rat at an older age, suggesting antiaging activities of TP. Consistent with these findings, we had previously reported that TP intervention led to a reduction in NF-*κ*Bp65 expression and an increased level of Nrf2 in colorectal cells of the rats treated with azoxymethane (AOM) [24]. Moreover, Hung et al. showed that lycopene administration suppressed the activation of NF-*κ*B and expression of intercellular adhesion molecule 1 (ICAM-1) through reducing TNF-*α* human endothelial cells [44]. It is of note that Yang et al. also reported that lycopene induced Nrf2 activation, resulting in enhanced expression of its downstream target, heme oxygenase-1 [45]. A limitation of our study is that we have not measured downstream molecular events such as hepatic expression of TNF-a and IL-6. We also did not examine gene expressions such as heme oxygenase-1, glutathione-S-transferase, and NAD(P)H:quinone oxidoreductase-1 to support the activation of Nrf2 signaling.

The mTOR signaling pathway has been shown to act as a key regulator of aging. mTOR plays an important role in the control of protein synthesis through phosphorylation of 4E-BP1 and S6K1 [16]. In addition, mTOR also modulates the lipid biosynthesis, autophagy, glucose metabolism, and mitochondrial function during aging [46]. In the present study, we showed that the phosphorylated levels of mTOR, 4E-BP1, and P70S6K1 in the hepatocytes of 77-week-old rats are higher than those of younger control animals (10-week-old). It is of note that TP supplementation significantly reduced the phosphorylation of mTOR, 4E-BP1, and P70S6K1 proteins in the hepatocytes of rats at the age of 77 weeks. Our data provide the first convincing evidence that TP modulates the mTOR signaling in the liver during the aging process. Moreover, we demonstrated that the reduced activity of the mTOR pathway in the livers of rats fed a diet supplemented with TP resulted in a direct correlation with decreased glucose and lipid profiles, suggesting that TP supplementation improves the glucose and lipid metabolism in rats through inhibition of mTOR signaling. These findings were consistent with previously published results on the association of decreased mTOR activity with reduced levels of glucose and lipid [46, 47]. Furthermore, our laboratory has previously shown that lycopene reduced the diethylnitrosamine-induced elevation in phosphorylation of mTOR, 4E-BP1, and P70S6K and expression of protein kinase B in rats, supporting our findings in the present study [48]. Ip et al. also reported that lycopene supplementation in BCO2-knockout mice resulted in suppression of oncogenic signals, including Met mRNA, *β*-catenin protein, and mTORC1 activation, which was associated with increased hepatic microRNA (miR)-199a/b and miR214 levels [49].

In conclusion, the present study demonstrates that TP supplementation has favorable implications for aging in healthy rats. Although 77-week-old rats are not that old, perhaps only middle-aged, but compared to young rats, they already have significant differences in the parameters measured. We showed that TP reduced liver damage and improved age-associated inflammation and oxidative stress through the inhibition of NF-*κ*B and mTOR pathways and activation of Nrf2 signaling. In addition, TP supplementation resulted in amelioration of metabolic parameters. Our findings provide strong support for future studies with TP supplementation in prevention of aging-related diseases. In fact, in a recently published study, Li et al. showed that tomato powder supplementation inhibits hepatic steatosis and inflammation through restoring SIRT1 activity, which is closely associated with aging [50].

## Figures and Tables

**Table 1:** Effects of TP supplementation on serum biochemical parameters in healthy rats.

Biochemical parameters	Groups (age at examination)				*P*<
	10 weeks old		77 weeks old		
	Control	TP	Control	TP	
Glucose (mmol/L)	3.85 ± 0.40^c^	3.81 ± 0.32^c^	5.24 ± 0.61^a^	4.19 ± 0.47^b^	0.01
T-C (mmol/L)	1.74 ± 0.13^c^	1.67 ± 0.26^c^	4.07 ± 0.35^a^	3.46 ± 0.39^b^	0.006
TG (mmol/L)	0.66 ± 0.05^c^	0.59 ± 0.03^c^	1.62 ± 0.05^a^	1.10 ± 0.02^b^	0.001
AST (U/L)	123.75 ± 7.21^c^	116.00 ± 8.92^c^	215.31 ± 11.51^a^	186.42 ± 9.64^b^	0.003
ALT (U/L)	43.18 ± 4.30^c^	42.5 ± 5.7^c^	85.61 ± 6.57^a^	68.20 ± .6.29^b^	0.001
BUN (mmol/L)	4.39 ± 0.23^c^	4.43 ± 0.62^c^	7.97 ± 0.31^a^	6.13 ± 0.55^b^	0.001
Cre (*µ*mol/L)	12.21 ± 0.96^c^	12.07 ± 1.23^c^	17.52 ± 1.11^a^	14.36 ± 1.29^b^	0.001

T-C: total cholesterol; TG: triglycerides; AST: aspartate aminotransferase; ALT: alanine aminotransferase; BUN: blood urea nitrogen; Cre: creatinine. The data are shown as the mean  ±  standard error. Mean values within a row with different superscript letters were significantly different at *P* < 0.05. ^a^Significant difference with TP for old rats (*P* < 0.05). ^b^Significant difference with control for old rats (*P* < 0.05). ^c^Significant difference with control and TP for young rats (*P* < 0.05).

**Table 2:** Effects of TP supplementation on serum and liver MDA levels and liver antioxidant enzymes in healthy rats.

Markers	Groups (age at examination)				*P*<
	10 weeks old		77 weeks old		
	Control	TP	Control	TP	
Serum MDA (nmol/mL)	1.31 ± 0.06^c^	0.97 ± 0.08^d^	2.76 ± 0.09^a^	1.83 ± 0.06^b^	0.003
Liver MDA (nmol/mg protein)	4.60 ± 0.15^c^	3.16 ± 0.21^d^	7.72 ± 0.56^a^	5.50 ± 0.22^b^	0.001
Liver SOD (U/mg protein)	184.07 ± 5.50^b^	234.48 ± 6.99^a^	123.21 ± 8.35^d^	157.52 ± 8.29^c^	0.001
Liver CAT (U/mg protein)	265.88 ± 11.30^b^	289.51 ± 9.16^a^	204.38 ± 7.63^d^	226.43 ± 9.19^c^	0.008
Liver GPx (U/mg protein)	33.26 ± 1.10^b^	46.20 ± 1.33^a^	15.55 ± 1.81^d^	24.38 ± 0.93^c^	0.01

MDA: malondialdehyde; SOD: superoxide dismutase; CAT: catalase; GPx: glutathione peroxidase. Data are the standard error of the mean (SEM). Mean values within a row with different superscript letters were significantly different at *P* < 0.05.

## Data Availability

The data used to support the findings of this study are available from the corresponding author upon request.

## Abbreviations

4E-BP1: Eukaryotic translation initiation factor 4E-binding protein 1
AMPK: AMP-activated protein kinase
CAT: Catalase
EGCG: Epigallocatechin gallate
FOXO: Forkhead box O
ICAM1: Intercellular adhesion molecule 1
IL-6: Interleukin 6
GPx: Glutathione peroxidase
MDA: Malondialdehyde
mTOR: Mammalian target of rapamycin
NF-*κ*B: Nuclear factor-*κ*B
NGF: Nerve growth factor
Nrf2: Nuclear factor erythroid 2-related factor 2
PI3K: Phosphatidylinositol 3-kinase
ROS: Reactive oxygen species
S6K: S6 kinase
SOD: Superoxide dismutase
TNF-*α*: Tumor necrosis factor-*α*
